# Acknowledgment to Reviewers of *Genes* in 2021

**DOI:** 10.3390/genes13020326

**Published:** 2022-02-10

**Authors:** Genes Editorial Office

**Affiliations:** MDPI AG, St. Alban-Anlage 66, 4052 Basel, Switzerland

Rigorous peer-reviews are the basis of high-quality academic publishing. Thanks to the great efforts of our reviewers, *Genes* was able to maintain its standards for the high quality of its published papers. Thanks to the contribution of our reviewers, in 2021, the median time to first decision was 19 days and the median time to publication was 36 days. The editors would like to extend their gratitude and recognition to the following reviewers for their precious time and dedication, regardless of whether the papers they reviewed were finally published:
Aakrosh RatanKazuhiko ImaizumiAaron DickeyKazuhiko KotaniAaron ReinkeKazuhiro SakuradaAashish SoniKazuki KurimotoAbdelhafid BendahmaneKazuo WatanabeAbdo MahliKazuo YudohAbdul NasirKazushi AotoAbdulqader JighlyKazuyuki NumakuraAbheepsa MishraKehua WangAbhijeet BakreKei FujiwaraAbhijeet SonawaneKei IidaAbhijit RathKei TobiumeAbhinandan PatilKeiichi IshiharaAbhishek ChatterjeeKeiichi TakataAbhishek KulkarniKeisuke NagaiAbhishek LunagariyaKeisuke YanagisawaAbhishek SrivastavaKeith A. CrandallAcaimo Gonzalez-ReyesKeith MartinAchille IolasconKeith WeaverAdam CawleyKejian WangAdam PyzikKelly WilliamsAdam Thil SmithKen StedmanAditi GurkarKen TakasawaAditya ShreenivasKengo SatoAdnan IsmaielKenichi TanakaAdnan Shami ShahKenji IkeharaAdrià ArboixKenneth KraemerAdrian BrennanKensaku SakamotoAdrián Rodríguez-ContrerasKent M. ReedAdrian RothenfluhKenta NakaiAdrianna SkonecznaKern-Zdanowicz IzabelaAdriano Jimenez-EscrigKerry SmithAdriano MagliKerryn ElliottAdriano TagliabracciKerstin MeyerAfsana HossainKeshava MysoreAgata FiumaraKeun HurAgata KlejmanKeunje YooAgata Leońska-DuniecKevin ChildsAgata TyczewskaKevin GlintonAgnès Desroches-CastanKevin KennaAgnieszka BanasKevin KieslerAgnieszka FornalKevin SilversteinAgnieszka HerosimczykKevin VaughanAgnieszka Jagiełło-GruszfeldKevin YangAgnieszka Lecka-AmbroziakKhaled AzizAgnieszka MostowskaKhushboo MunirAgnieszka MroczekKijin KimAgnieszka RygielKimberly RowlandAgnieszka Siomek-GoreckaKinga GawelAgnieszka Skalska-KamińskaKinga LinowieckaAgnieszka SzczepekKira MakarovaAgnieszka TudekKira TaylorAhmad OmarKira VeleyAhmed ElaswadKirill AntonetsAhmed GadKirill AzarinAi LuongKirk CammarataAideen McInerney-LeoKirk McManusAinhoa Robles-MezcuaKirk StephensonAisha SyedaKirsten HansonAjay AshokKitanaka JunichiAjay PalaganiKitty VijverbergAjaz LoneKiyoshi MasudaAjla WastiKlára KosováAkane KubotaKlaudia BrixAkanksha SinghKlaus DieterichAkikazu SakudoKlaus GaseAkiko MaedaKlaus JungAkinori MiyashitaKlaus WimmersAkio MiyaoKlaus-Peter GötzAkira IshikawaKlaus-Peter LeschAkira NifujiKnut RudiAkira SatoKoichi TakimotoAkira ShinoharaKoji SatoAkis PapantonisKoki FujisakiAladdin HamwiehKoldo Garcia-EtxebarriaAlain PerretKonrad HuppiAlakananda DasKonstantin KhrapkoAlaknanda MishraKonstantin PanovAlan ArchibaldKonstantinos KompotisAlan DeCherneyKonstantinos KoudounasAlan F. ScottKonstantinos SousounisAlan PittmanKorkut UlucanAlan WarrenKornel KasperekAlanna StrongKosagi RaoAlbena JordanovaKostas MathiopoulosAlbert BeckerKota WatanabeAlbert JeltschKozak GenevieveAlbert JordanKress WolframAlberto AcquadroKrinio GiannikouAlberto CesaraniKris ChristensenAlberto Fernández-MedardeKristaps JurjansAlberto VilaKristiaan Van Der GaagAlbrecht ReichleKristina KuhbandnerAldo DonizettiKristof De SchutterAldo GrefhorstKristopher SilverAlejandro GarantoKrisztián FrankAlejandro MoralesKrisztina BelaAlejandro VaqueroKrzysztof AndresAleksander SikorskiKrzysztof KowalAleksandr SahakyanKrzysztof SzczałubaAleksandra DunisławskaKui ZhangAleksandra PavlovićKuldeep AttriAleksandra Piechota-PolanczykKuldeep GuptaAleksandra Wojnicka-PółtorakKumiko Ui-TeiAles KovarikKwang LimAleš MaverKwangsic JooAlessandra TesserKwang-Soo ShinAlessandro AchilliKyle McElroyAlessandro BarbonKyle WellbandAlessandro FeolaKylie CairnsAlessandro GrapputoKyu-Sang LimAlessandro IannacconeLada Lukić-BilelaAlessandro NataliniLadislav HavelAlessandro PoliLafon-Hughes LauraAlessandro PolizziLaila ZikoAlessia CappelliLakkakula SatishAlessia FaggianLamia BoukhibarAlessia IndrieriLan XiongAlessio BranchiniLarissa KotelevetsAlessio Di FonzoLars Allan LarsenAlex ClopLars FeukAlex FeltusLászló Kozma-BognárAlex PostmaLaszlo PatthyAlex TrollopeLaura AudìAlex VargheseLaura BaldoAlexa SiddonLaura Cervera-CarlesAlexander AwgulewitschLaura CrisponiAlexander BerezinLaura EadieAlexander BotzkiLaura GhigliottiAlexander GombertLaura HeathAlexander GrahoferLaura Marie ThorntonAlexander IonidesLaura MarroquiAlexander KarpukhinLaura ParmaAlexander KleschevnikovLaura PezzoliAlexander KovalchukLaura SackAlexander KrivoruchkoLaura SánchezAlexander MankinLaura SwanAlexander NeefLaura ZingarettiAlexander OtahalLauren LaboissonniereAlexander ParedezLauren WeissAlexander S. GraphodatskyLauren WhiteAlexander SermyaginLaurent MetzingerAlexander TyakhtLaurent TiretAlexander V. EreskovskyLavanya DampanaboinaAlexander Van ParysLawrence RothblumAlexander VershininLea BennettAlexandra KretzLeandro Pereira-DiasAlexandra StanleyLee Hwan-youngAlexandra ZakharenkoLeena ShewadeAlexandre GrondinLefkothea KarapetsiAlexandre MarchandLei DingAlexandre PeléLei GuoAlexandre ReymondLei WangAlexandros ProtonotariosLei WeiAlexessander Couto-AlvesLeif KirsebomAlexey BurdennyyLeila YoussefianAlexey MeshkovLela BuckinghamAlexey PolonikovLeo VeenmanAlexey TesakovLeonardo AriasAlexis MichenetLeonardo Emberti GialloretiAlfonso Benítez-PáezLeopold EckhartAlfred HandlerLeopoldo IannuzziAlfred RocaLeslie MyattAlfred SzmidtLeszek KarlińskiAlfredo BruscoLeticia SmithAlfredo CasanovaLev PrasovAlfredo CaturanoLeyre MadariagaAlfredo Cruz-RamirezLi YiAlfredo PulvirentiLi ZhangAli AghdassiLi ZhaoAli AhmadLiangjiang WangAli KaissiLiangxue LaiAli KhansariLibor KrásnýAli MovahediLidia LarizzaAli RazaLidia ZiółkowskaAli Salehzadeh-YazdiLiem PhanAlice BongLiesbeth VossaertAlice DavidsonLige TongguAlice LacinyLikun DuanAlice VercellinLikun WangAlicia RoqueLily WangAlicja DolzblaszLim JanghooAlicja PacholewskaLina YoussefAlicja Stachelska-WierzchowskaLinda ReisAlina AvanesyanLindsey PerkinAline Rangel-PozzoLindsey Van HauteAlise PonseroLine KesselAlison MeynertLing WangAliza P. WingoLingfeng KongAlja PaskaLingling AnAllen ByrdLingxiao HeAllison D. EbertLinjie WangAllison FisherLinley JessonAlmudena Espin PerezLionel BenardAlmudena FernándezLiping ChungAlok ArunLiping GuAlok SharmaLisa PetrellaAlsu SaifitdinovaLisa RileyAlvaro Gallego-MartinezLisa WiesmüllerAlvaro Montesinos JovenLisbeth TranebjærgAlwin KrämerLishuang ShenAmalio TelentiLiu YiAmanda J. MyersLiujiang SongAmanda KowalczykLivia GaravelliAmanda LindholmLivia López-NoriegaAmanda WasylishenLiwei WuAmandeep SinghLixi JiangAmandine MorettonLiye WangAmelia CasamassimiLiza GershonyAmer DababatLizhong WangAmir FallahshahroudiLjiljana KuzmanovicAmit KumarLliljana PetrovskaAmit NahumLluís Ribas de PouplanaAmit SinghLogan ColeAmjad KhanLoganathan PonnusamyAmlan PatraLoic LemonnierAmy FindlayLorena Joga-ElviraAmy Jayne McKnightLorena RuizAmy MessersmithLorena SaelicesAmy PowellLorenzo CaputiAna Barletta FerreiraLorenzo FarinaAna CampilhoLorenzo PaceAna Carvajal UreñaLorenzo RossiAna ConesaLotta Granholm-BentleyAna Do PaçoLounés ChikhiAna EsquifinoLourdes SerranoAna Furlanetto PachecoLouxin ZhangAna GorostidiLubica DudakovaAna Latorre-PellicerĽubica ĎudákováAna OliveiraLubo ZhangAna PereiraLuboš VostrýAna Sánchez-HerasLuca AmbrosinoAnália LourençoLuca BeltrameAnamaria JurcauLuca Falzone‪Anand SinghLuca SpieziaAnantha HarijithLuca SteardoAnastas GospodinovLuca TeseiAnastasia MalekLucas Dos Santos DiasAnastasios GkountakosLucas LeclèreAnatoliy DrozdovLucas SalasAnatoly TiulpakovLucia MicaleAnbupalam ThalamuthuLucia MuggiaAnca MacoveiLucia NatarelliAnca SimionescuLucia ZifcakovaAnda GligaLuciana CaenazzoAndaleb KholmukhamedovLucio BertarioAnders BergstromLucio MangoAnders JørgensenLudmila Danilowicz-SzymanowiczAndre TavaresLudmila Prokunina-OlssonAndrea Ascoli MarchettiLudwig WagnerAndrea BragaglioLuigi CarboneAndrea FusoLuigi De MasiAndrea GelemanovićLuigi DonatoAndrea GhiroldiLuigi MinafraAndrea HeinzLuigia De FalcoAndrea L. BadenLuigia StucciAndrea McWhorterLuis BotoAndrea PellagattiLuis CaravacaAndrea RandoLuís GalesAndrea RiccioLuis GaliettaAndrea RonchiLuis LeitãoAndrea Sanchez ValletLuis Lopez MauryAndrea ScharfLuis LugonesAndrea SchraderLuis NagaiAndrea SchubertLuís PintoAndrea ŠoltýsováLuís RatoAndrea TangherloniLuis RodriguezAndrea UrbaniLuis VaronaAndreas BeckLuisa CircelliAndreas BorchelLuisa GarofaloAndreas ChiocchettiLuisa PolitanoAndreas G. ChiocchettiLuiz Santana-Da-SilvaAndreas HadjisavvasLukačová ZuzanaAndreas HochwagenLukáš KratochvílAndreas SchulzeLukas MuellerAndreas ScorilasLukas VargaAndreas TzschachLuke JacobusAndrei RodinLuonan ChenAndrei SurguchovLuo-Yang DingAndreia AmaralLu-Sheng HsiehAndreja Trojner-BregarLusine DemirkhanyanAndrés BervilléLuye QinAndrés CortésLynn BushAndrew ButlerLynsey HallAndrew Jan WaskiewiczLyubov SalnikovaAndrew MallettM. AlonsoAndrew MannM. Andres BlancoAndrew McKechanieM. IqbalAndrew NelsonM. Michael GromihaAndrew OzgaMaarten KamermansAndrew PikeMaciej GeremekAndrew RouthMaciej SzymańskiAndrew SchurkoMaciej TarnowskiAndrew SinclairMaciej WnukAndrew VealeMaddalena MartellaAndrew WolffMadelyn GillentineAndrey FrolovMaëlle PannetierAndrey HöglundMagali CasanovaAndrey MarakhonovMagdalena BogutaAndrey SavchenkoMagdalena CalAndrey ShirakMagdalena KolendaAndrii FatiukhaMagdalena SimlatAndriy BilichakMagdalena StasiakAndrzej BajguzMagdalene J. SeilerAndrzej CiechanowiczMahima SinghAndrzej JerzmanowskiMahmoud HuleihelAndrzej PawlikMaija CastrenAndrzej PławskiMainá BitarAndrzej TeisseyreMaja BoczkowskaAndrzej ZachwiejaMaja MatulićAndy Dr. BaileyMajed WakidAnelia HorvathMajid MomenyAnestis KarkanisMakoto HosoyaAneta AleksovaMaleszewski MarekAneta OlszewskaMałgorzata DudaAnette Ramm-PettersenMałgorzata GrzankaAngel AlvarezMałgorzata Polz-DacewiczAngel CarracedoMałgorzata RydzaniczAngela C. HirbeMałgorzata SułkowskaAngela FleischmanMałgorzata TokarskaAngela Garcia SanchezMałgorzata TokłowiczAngela KruseMalgorzata WasniewskaÁngela PrudencioMałgorzata WorwagAngelika SchniekeMalgorzata ZatykaAngeliki AsimakiMali Salmon-DivonAngelina NunziataManee ManeeAngelo FacchianoManes KonstantinosAnica KlockarsManfred KayserAnikó UjfalusiManmeet RawatAnil KadegowdaManoël ProuteauAnindita BasakManoj KandpalAnisa RibaniMansi SrivastavaAnita BhattacharyyaMansuck KimAnita Cybulska-KlosowiczManu KumarAnita GiglioManuel A. Garrido-RamosAnita MehtaManuel Alvarez-RodriguezAnja EhrhardtManuel CurtoAnja JoachimManuel IrimiaAnja Katrin BielinskyManuel Vera RodríguezAnja WeiseManuela PellegriniAnke BeckerManuela PrioloAnket SharmaMao-Lun WengAnkit SabharwalMaosen WangAnlu ChenMara ZilocchiAnn AtanganaMaranke KosterAnna BersanoMarc BartoliAnna BogdanovaMarc LibaultAnna ChaloupkaMarc PoirotAnna Ciemerych-LitwinienkoMarc ViaAnna CollMarc Woodbury-SmithAnna De BenedettoMarcella AvondoAnna EiringMarcella SabinoAnna EnglerMarcella ZollinoAnna Fernandez-FalguerasMarcellino MondaAnna GiammancoMarcello MezzasalmaAnna GrimaldiMarcello NicetaAnna HurstMarci SontagAnna J. KorzekwaMarcia ManterolaAnna Jakubska-BusseMarcial EscuderoAnna JurasMarcin LisAnna KlepikovaMarcin NicosAnna KukekovaMarcin OzarowskiAnna LinderholmMarcin PorębaAnna LinkiewiczMarco CatoniAnna M. MastrangeloMarco EmiliAnna MoonMarco SpadaAnna PanyushkinaMarco ToloneAnna Rita RossiMarco ZeppieriAnna RossiMarcos Díaz-GayAnna SimonsenMarcos DuarteAnna SlawinskaMareike RascheAnna TorgashevaMarek ŁukaszewiczAnna VaskuMarek PieszkaAnnalisa MarcheseMarek SanakAnne BrownMarek SawczukAnne E. KwitekMargaret ReynoldsAnne KowalczykMargarida Gama-CarvalhoAnne NossentMargarida MatosAnne Schaafsma SchaafsmaMargarita GeorgievaAnne-Christine PlankMargarita TodorovaAnne-Laure FerchaudMari PhilipsAnne-Sophie ArmandMari Vázquez-BorregoAnnette BeckerMaria Aguado MolinaAnnick DuboisMaria AnisimovaAnnie HinikerMaria ArnedoAnnie RobicMaria BabakAnnik GmelMaria BarallobreAnshika JainMaria BaturovaAnshu AlokMaría BordesAnte IvankovićMaria Bueno MarinasAnthony ArnoldMaria C. DeRosaAnthony BleyerMaria CaligoAnthony FordMaria CardoneAnthony VecchiarelliMaria CataniaAntoine Bridier-NahmiasMaria ChahrourAntoine RimbertMaria CosciaAnton I. PetrovMaría EstebanAnton LennikovMaria FernándezAntonella CamaioniMaria FousteriAntonella IzzoMaria GiansantiAntonella MinutoloMaría GomezAntoni Riera-MestreMaria Gómez-CasaresAntonia FollenziMaría HerreroAntonia MaioMaría José AranzanaAntonia NoceMaria KorominaAntonina ArgoMaría LallenaAntonino NeriMaria Lanfranco GallofreAntonino TuttolomondoMaria LongeriAntonio AmorimMaria LopesAntonio CaruzMaria Luisa ChiusanoAntonio FiguerasMaria MarcheseAntonio GiordanoMaria MarketouAntonio González ArizaMaria Moreno-VillanuevaAntonio Gonzalez-BulnesMaria MunizAntonio González-MartínMaria NittiAntonio GranellMaria OczkowiczAntonio José Piantino FerreiraMaria OgielskaAntonio LepeddaMaría Padín-IruegasAntonio ManentiMaria PapatsirouAntónio MonteiroMaria PiccioneAntonio NovelliMaria PireddaAntonio OrlacchioMaria PiulachsAntonio PercesepeMaria PrünsterAntonio SalvaggioMariá RabaglinoAntónio Sebastião RodriguesMaria SharakhovaAntonio SermekMaria SollerAntonio Serrato-CapuchinaMaria StrazzulloAntonio VentosaMaria ValievaAntonio VillaloboMaria ValorosoAntony CougnouxMaria VarelaAntony KaspiMaria VieiraAparna SwaminathanMaria VivoAphrodite TsaballaMaria VorontsovaApril HillMaria-Jose TrucoArcadi Navarro CuartiellasMariamena ArbitrioArgelia Medeiros-DomingoMariangela De RobertisAriadne Hager-TheodoridesMariangela SabatellaArianna MazzoliMarianna AprileArianna VigniniMarianne LundAriel KushmaroMariano Loza-CollArijita SarkarMarianthi GeorgitsiArimatias RaitioMariarita BrancaccioArindam BhattacharjeeMariarosa Anna Beatrice MeloneAristeidis KatsanosMaribel Forero-CastroArlindo OliveiraMaribel Quezada-FeijooArmando CaballeroMarie AbitbolArmin KrausMarie AltmanováArnab BasuMarie BaudetArnab GhoshMarie BeaumontArnaud CarrierMarie CastetsArne LudwigMarie GaineArnold GrünwellerMarie-José GourmansAron GeurtsMariella CuomoArpan AcharyaMarie-Louise KampmannArrigo CiceroMarika KaakinenArsalan YousufMarina AscunceArsen DotsevMarina BakayArtem LisachovMarina ColombiArtem NedoluzhkoMarina FelisbinoArthur BergMarina FericArthur FoulonMarina GottikhArthur ZalevskyMarina Ramal-SanchezArtur BossowskiMarina ZytsarArturo AndradeMario Burgos-AcevesArturo BevilacquaMario CáceresArturo OrlacchioMárió GajdácsAsher OrnoyMario Martínez-NúñezAshok PatowaryMario VenturaAshutosh PandeyMario ZuritaAsim EjazMariola JaniszewskaAslihan TemelMariola SłowińskaAsmi ChakrabortyMariolina GullìAsp JuliaMariona BustamanteAssaf DistelfeldMarisa BartolomeiAssunta SellittoMarius ZahanAstrid Adarmes-GómezMariusz BerdyńskiAsunción ContrerasMariusz HartmanAtashi SharmaMariusz SkowronskiAthanasios ZervasMarjorie JonesAtif AdnanMark BerneburgAtsuo YoshidoMark BoldinAtsushi ShibataMark BuchheimAtsushi WatanabeMark C. FieldAttila KeresztMark C. HirstAttila SándorMark DodsonAttila ZsolnaiMark EvansAtul PandeyMark HarrisonAtul PranayMark HedglinAugustin OfiteruMark HelmAugusto SolaMark HoogendijkAureliano BombarelyMark PepinAurelio Luna MaldonadoMark RanekAurora NedelcuMark ZanderAurora Ruiz-HerreraMarko JakopovicAurore CarréMarko KnollAussie SuzukiMarkus BischoffAvelino Álvarez-OrdoñezMarkus DuechlerAvi Orr-UrtregerMarkus J. HerrgårdAvinash ShanmugamMarkus KallerAvinash WaghrayMarsha WallaceAwawing AndongmaMarta Carrillo-PalauAxel SchambachMarta CuadrosAyman EL NagarMarta Hałas-WiśniewskaAyse MutluMárta MedveczAzza MohamedMarta Migocka-PatrzałekAzzurra StefanucciMarta Obara-MichlewskaBabu ValliyodanMarta PuigBailey EngleMarta RieraBaldassarre MartireMarta Sánchez-CarbayoBálint NagyMarta WłodarczykBallios Brian G.Marthe-Susanna WegnerBaosheng ZengMartien GroenenBaoye HeMartin BartasBarbara ArbeithuberMartin BeranekBarbara BardoniMartin ČertnerBárbara HenriquesMartin ChadwickBarbara JenkoMartin FellnerBarbara KahlMartin FeulnerBarbara MajelloMartin IrestedtBarbara MolesiniMartin KuhlwilmBárbara PalazonMartin LaimerBarbara PicconiMartin McKibbinBarbara SozańskaMartin PestaBarbara WallnerMartin ProchazkaBart PannebakkerMartin RöserBartłomiej NiespodzińskiMartin ŠimonBartosz KempistyMartin SztachoBasar CenikMartin VingronBassam ElgamoudiMartin ZiegerBastien LlamasMartina Johnson PokornáBeáta HubkováMartina KroppBeata PepłońskaMartina RossiBeata SitkowskaMartina SilvestriBeate Brand-SaberiMartina TorricelliBéatrice BocquetMartina ZappaterraBeatriz Gutierrez-GilMartine BesouwBedabrata SahaMartine CrosetBee TanMartine Doco-FenzyBeiyan ZhouMarvin MillerBen ZonneveldMarwa HANAFIBeniamino TrombettaMary MajBenjamin DelpratMaryam ArdalanBenjamin HorwitzMarymegan DalyBenjamin LoMarzena LewandowskaBenjamin MederMarzena MaćkowiakBenjamin N. SacksMarzena OlesinskaBenjamin OnyeaguchaMarzia OgnibeneBenjamin ShawMarzieh SalimiBenoit ArveilerMasafumi KameyaBenoît LacombeMasahiko AjiroBenoît PalancadeMasahiro HayashiBerk BurguMasahiro KanedaBernard S. LopezMasaki NaganeBernardo BertoniMasako SuzukiBernd LenzMasami MorimatsuBernd WeisshaarMasashi MizuguchiBernd WissingerMasataka SuzukiBernhard HerrmannMasatoshi MatsunamiBernt PoppMasatoshi NomuraBert DriessenMasayuki TakedaBertrand PainMasfique MehediBethany RedelMassimiliano CardinaleBetty GardieMassimiliano ChettaBiagio PalmisanoMassimo Di GiulioBianca ColonnaMáté VargaBianca HabermannMateusz MaciejczykBianca WaudMateusz MajdaBidisha RoyMateusz MolonBimal ChaudhariMateusz RytelewskiBin GuMathew AlexanderBin YuMathew BloomfieldBina JoeMathieu QuinodozBinbin LiuMathieu SertorioBing YangMathilde CarpentierBirgit LorenzMathilde CausseBirgitta GläserMatias BustosBiruk FeyissaMatias MorinBiswajit PadhyMatias WagnerBjarni J. VilhjalmssonMatt PowellBjörn VoßMatteo BarberisBlake SweeneyMatteo CalcagnileBlanca BarqueraMatteo CerriBlazej RubisMatteo ChiappediBo LeiMatteo Dal FerroBoas PuckerMatteo MartinaBodhisattwa BanerjeeMatteo ZampiniBogdan DorofteiMattheos KoffasBoguang YangMatthew BensonBoguslawa LuzakMatthew BernsteinBohuslav JanousekMatthew BrownBojan ŽunarMatthew ButchbachBolesław KalickiMatthew ChristmasBoleslaw KarwowskiMatthew HarmelinkBonnie BaxterMatthew OrdidgeBoris SlobodinMatthew StrubBorut PeterlinMatthew WieduwiltBourgeois CyrilMatthew YousefzadehBožena Ćurko-CofekMatthias MäurerBrad BinderMatthias ThalmannBradley KellerMatthias VoggBradley OlsonMatthias WuttkeBrankica MravinacMatti JauhiainenBregje Van BonMaurice LingBrenda MurdochMauro AlaibacBrenda OppertMauro GiuffrèBrendan HoustonMauro PetrilloBrendan LoftusMax HübnerBrendan ReidMaxime PichonBrenton Von Takach DukaiMayumi YoshiokaBrian C. VerrelliMazdak SalavatiBrian CharlesworthMazène HochaneBrian ChungMd. UddinBrian ReidMedhat MahmoudBrian T. WilhelmMegan PovelonesBrian VerrelliMehdi PiroozniaBridget AylwardMehdi SoltaniBrígida PinhoMehmet GültasBrigitte SchlegelbergerMeichi ChangBrijesh AngiraMelanie CareBruce BudowleMelanie PercyBruce CohenMelanie WaldenbergerBruce ShapiroMelissa LittleBrunella FrancoMelita Vukšić PolićBruno FiondaMendell RimerBruno González-ZornMeng MaoBrutch NinaMeng WangBryan HaradaMeng WuBryan MowryMeng ZhuBrynn H. VoyMerel PostemaBurkhard BergerMeriel JonesC. A. M. J. J. Van den HondelMerike JõesaarCaihong WeiMerita XhetaniCaitlin HudacMervyn ThomasCallum DonnellyMgr. PrerostovaCalvin O. QualsetMichael BenedikCamelia OroianMichael CalcuttCamelia SavaMichael CampanaCameron BrackenMichael CowleyCamilo ToroMichael CunninghamCandela HernándezMichael FayCarl ClassenMichael FieldCarla BizzarriMichael GänzleCarla CalòMichael HildebrandCarla EmilianiMichael HofreiterCarla FontanaMichael HölzelCarla Solé CañadasMichael J. PalladinoCarla VerriMichael KempCarlo AlviggiMichael LaydenCarlo CasaliMichael LeeCarlo MaleyMichael McClellandCarlo Maria Di LiegroMichael NeugentCarlo PernoMichael PortelliCarlo SalaMichael SchutzCarlo TicconiMichael StaceyCarlo V. CatapanoMichael TrakselisCarlos A. CanchayaMichael TrembleyCarlos BarataMichael WhiteCarlos CanchayaMichael WhitfieldCarlos Contreras-MartelMichael ZasloffCarlos FarinhaMichaela BotiCarlos FernandesMichaela MedováCarlos LoncomanMichail KlontzasCarlos MinuttiMichail RovatsosCarlos Rodríguez OsorioMichail T. RovatsosCarlos SuarezMichal HetmanCarme FabregaMichał KulusCarmelo LuciMichal SchweigerCarmen CadillaMicheal McLellanCarmen FasoMichel ArthurCarmen GallasMichel RavelonandroCarmen Guillén-PonceMichela CasellaCarmen RinaldiMichela DentiCarol ImbrianoMichela SugniCarole CreuzenetMichele PinelliCarolina BonillaMichele RagazzoCaroline DenesvreMichele RubiniCaroline Junker MentzelMichelle AxfordCaroline MauveMichelle McClementsCaroline MonteilMiguel AndradeCaroline Schluth-BolardMiguel ArenasCarpintero-Fernandez PaulaMiguel Baltazar-SoaresCarrie FinnoMiguel BotellaCastrense SavojardoMiguel Cacho TeixeiraCatalina SantiagoMiguel Castelo-Branco SousaCatalina Valdés-BaizabalMiguel ConstanciaCatarina BrancoMiguel De VegaCatherine BrownsteinMiguel EstravísCatherine CollinsMiguel Leiva-BrondoCatherine GrueberMiguel Martin-CaraballoCatherine Van RaamsdonkMiguel Vicente-ManzanaresCatherine YzydorczykMihaela PerteaCatia ScassellatiMiho TakemuraCatriona KellyMikael OscarsonCatriona ThompsonMikhail GantsevichCécile RaynaudMikhail ShepelevCecilia AmbrosiMikko FrilanderCecilia BattistelliMikko TurunenCecilia VecoliMilad MiladiCees Van DeursenMilan KubiatkoCelia CantinMilena RizzoCelia HardingMiloš MacholánCélia SoaresMilos PjanicCéline BonMiloš VittoriCeline LevesqueMiloslav SandaCeline ReisserMin HongCenk SahinalpMin KimCesar Arrese-IgorMin SongCesar Cortez-RomeroMin YouChaeyoung LeeMina KavianiChanchal MandalMin-Chih ChengChandler WalkerMing HsiehChandra BathulaMinggui PanChandra PareekMingli WangChandrasekhar KottakotaMingyi ZhangChandrima ShyamMiodrag GrbicChang LiMiriam BauwensChangmin SungMiriam ElbrachtChao WangMíriam Molina-ArcasChao-Wen LinMiriam SchmidtsCharles BellMiriam ZemanovaCharles Bou-NaderMiriam-Rose MenezesCharles HancockMirko ManchiaCharles Van PraetMiroslav PatrCharlotte AlstonMiroslav PlohlCharlotte HuberMirosław AndrusiewiczCharlyn PartridgeMirosław RatkiewiczChase SmithMirosław TyrkaChenchen ZhaoMiroslawa DabertChen-Chi WuMiruna MicheuChenglei LiMitchell HollandCheng-Ming ChuongMitchell Mark HollandChenjiang YouMitsuko FuruyaChethan SampathMiwako NishioChevalier ChristianMohamed AbdulHameedCheyenne TaitMohamed AliChia-Jung ChangMohamed SaadChian JongMohamed ZaiouChiara AiroldiMohammad AlkhatibChiara MagriMohammad NilforooshanChiara VillaMohammad RahmanChie TomikawaMohammad VatanparastChieh-Lin TengMohammed IlyasChien-Teh ChenMohammed KhanChin HsuMohan GuptaChin-Chuan HungMolnár Tamás GergelyChing-Ho ChangMomose TsuyoshiChinmay TikheMona BatishChisato KinoshitaMonica BorghiChitrala Kumaraswamy NaiduMonica BullejosChiu-Ping ChengMônica De MeloChong TengMonica MazzucatoChongkui SunMonica MencarelliChoongwon JeongMonica MiazziChris PlanqueMonika Dmitrzak-WęglarzChristelle LeungMonika HorváthováChristian BeltonMonika OłdakChristian ChevalierMonika PechhackerChristian HuberMonika Proszkowiec-WeglarzChristian PosberghMonika SkorupaChristian RoosMonika Stefaniuk-SzmukierChristian RudolphMonika UlamecChristian SchlöttererMontserrat CorominasChristian SchöferMorag LewisChristian SiadjeuMorgan SalmonChristian SiatkaMorouj Al-AjeeliChristiane BranlantMorris MaduroChristina BergeyMorten GjerstorffChristina Gerth-KahlertMostafa KhairChristina LaukaitisMotoki WatanabeChristina MertensMozheh ZamiriChristina RochusMrigendra RajputChristina SotiropoulouMubshar HussainChristina ZeitzMuck-Seler DoroteaChristine DillmannMuhammad BashirChristine HappelMuhammad KabirChristine HertlerMuhammad NomanChristine KayMuhammad RiazChristine KiireMuhammad TehseenChristine LerouxMuh-Shi LinChristo KoleMukesh SriwastvaChristoph GerhardtMulyoto PangestuChristoph GrunauMurat AycanChristoph PlassMusa HassanChristoph RieckMushriq AbidChristophe EscudéMyoung ParkChristopher BrandlMyung-Shik LeeChristopher CollinsNadege BondurandChristopher MakaroffNader AldewikChristopher PenkettNádia PintoChristopher PhillipsNagarajan RajuChristopher SauvageNam HoangChristos BazakosNan WuChristos GeorgiouNan YangChristos KontosNancy OlsenChristos NoutsosNanda RouthuChuankui SongNan-kai WangChun-Che ChangNao OtomoChunhong ChenNaomi PorretChun-Wei TungNaoomi TominagaCingel AleksandarNaoya KenmochiCinzia CiccacciNaoyuki KataokaClaire-Marie DhaenensNaoyuki TakahataClaude CharvetNaoyuki TsuchiyaClaude FerecNarayan RathClaudia CatarinoNarendiran RajasekaranClaudia DurantiNariman BattulinClaudia FalliniNaruo NikohClaudia FolliNasib QureshiClaudia KohlNatalia AraujoClaudia PföhlerNatalia KrawczynskaClaudia RicciNatalia RakislovaClaudio AfonsoNatalia RyczekClaudio BernardazziNatalia ShestakovaClaudio GrazianoNatalia ZinovievaClaudio LuchiniNatalie ForsdickClaudio QuilodránNatalie TwineClaus VoglNatalie WallisClemens ZwergelNatascha VukasinovicCleverson MatiolliNathalie BissonnetteClifford SteerNathalie CasseClint L. MillerNathalie OulhenClint MagillNathalie Van Den BergeClive EvansNathan ChrismasColin CampbellNathan JohnsonColleen Glyde JulianNathan PalmerColleen MayberryNathan ScudderConcepció MarinNathan SutterConcepción Vaquero LorenzoNati HernandoConcetta CrisafulliNatraj KrishnanConchi EstarásNatsuko ChibaCongBao KangNaveenchandra SuryadevaraCong-Jun LiNavneet MatharuConny Van Ravenswaaij-ArtsNazli McDonnellConstantinos DeltasNazmul HudaCorey NislowNeelakanta KanikeCornelia BraicuNeetha VellichirammaCornelia Van DuijnNeha NandaCorrado AngeliniNeha ShuklaCosmas NathanailidesNeil GreenspanCourtney BabbittNelson TangCraig McLachlanNemeth MarkCrispin MutshindaNeonila KononenkoCristian BonviciniNéstor García-RodríguezCristian CañestroNestor KippesCristian FioriNeveen SaidCristiano GarinoNicholas BoermanCristiano SimoneNicholas CliftonCristina CantaleNicholas HaleyCristina CozziNicholas HaywardCristina De Guzman StrongNicholas HobbsCristina González RavinaNicholas J. ShortCristina Mas-BarguesNicholas LarsonCristina OlivoNicholas MorffyCristina Sánchez-De-DiegoNicholas SmithCristina Vives CoboNick WarnockCristoforo PomaraNico M. Van StraalenCsaba ÉvaNicola ArmstrongCuong NguyenNicola Bougen-ZhukovCurie AhnNicola RoyleCynthia BradhamNicola TarantinoCyril RibeyreNicolae GicaDagmara KabzińskaNicolas ArnaudDahang YuNicolas BlouinDaisuke KageyamaNicolas DelhommeDale R. NyholtNicolas FaselDaljit SinghNicolas JacquierDamain HolsingerNicolas NassarDaman KumariNicolas PilonDamer BlakeNicolas RamozDamian CzarneckiNicolas SantiquetDamian GawelNicolas VerheyenDamian SuñerNicole CoufalDan GraurNicole FerraraDan MilbourneNidhi SharmaDaniel AbankwaNiels BrouwerDaniel BinzelNigel BurrowsDaniel BorregoNigel O’NeilDaniel CalameNigel WilliamsDaniel CronaNihay Laham-KaramDaniel Da Silva E SilvaNika PetrovaDaniel DuranNikola ŠtokovićDaniel FischerNikola Zlatkov KolevDaniel García SoutoNikolai AdamskiDaniel GeschwindNikolaos IoakeimidisDaniel HooperNikolay BarashkovDaniel J. WeisenbergerNilima PrakashDaniel JeffaresNils HallerDaniel MonaghanNina GašperovDaniel MorgansternNina Gunde-CimermanDaniel MouzoNing DingDaniel Ocampo DazaNing JiangDaniel PotaczekNishana MayiladumveetilDaniel RowleyNishitha PillaiDaniel SchriderNitesh MundDaniel VanekNobuhiko OkamotoDaniel VillagomezNoel RaynalDaniela CaccamoNoelia Martínez-SánchezDaniela Di BellaNoland MartinDaniela PalmaNoor KazimDaniela PiancatelliNóra BákonyiDaniela RosadoNora NockDaniela SerbanNorihiro SuginoDaniela UbertiNoritaka SaekiDaniele GarcovichNoriyasu HirasawaDaniele OrsucciNorman PaceDaniele VergaraNunzia MontuoriDanielius SerapinasNunzio CacciolaDanika BannaschNúria Coll-BonfillDanila CuomoNuria Torres-AguilaDanillo AlvarengaOctavian PopescuDanilo FintiniOctavio MartínezDanilo IannettaOdile LecompteDanon CardosoOier EtxebesteDanuta LoeschOihane Diaz De CerioDany BesteØivind BerghDany Domínguez-PérezOlaf BodamerDaochun KongOlaf NielsenDao-Qiong ZhengOlaf SommerburgDaotai NieOlaia Martinez IglesiasDarin ZertiOleg DenisenkoDario BalestraOleg TimofeevDario OjedaOleksandra TiapkoDario StrbenacOlga BlagovaDariusz GrzebelusOlga DolgovaDariusz KucharczykOlga PodgornayaDarpan ChakrabortyOlga PosukhDarren GriffinOliver RossbachDave WynneOlivera TopalovicDavid A. ChambersOlivia MajerDavid A. SimpsonOlivia VeatchDavid Alvarez-PonceOliviero CarugoDavid Baez-NietoOlli-Pekka SmolanderDavid Barros-GarcíaOluwatosin OluwadareDavid ButlerOmaththage PereraDavid C. SprayOmid FotouhiDavid CaramelliOmri WurtzelDavid DolivoOndrej SlabyDavid EideOrdan LehmannDavid FerrierOriana Lo ReDavid GaikpaOriol De BarriosDavid Gómez MatallanasOsagie IzuoguDavid HaymerOsamu GotohDávid HerczegOscar CampuzanoDavid HoldingOscar FantiniDavid IrwinOscar LaoDavid Lea-SmithOskar HaasDavid MacAlpineOsvaldo Bogado PascottiniDavid MarksOthmane MerahDavid MeyerholzOttmar DistlDavid N. CooperOttmar HerchenröderDavid OrrenOukseub LeeDavid ParryOyuna KozhevnikovaDavid RayÖzgen DenizDavid SibleyOzgur BayramDavid SibsonP. ChaseDavid SmalleyPablo CerdánDavid StaceyPablo CerroDavid SteffenPablo LibradoDavid SullivanPaige MundyDavid VermijlenPaloma MoránDavid WeisblatPamela LagaliDavide ArcanioloPamela RossoDavide SoglianiPamela TozzoDavina DerousPanagiotis KatsonisDawid GrzelaPanagiotis MoulosDayna DregerPankaj SharmaDe ChigbuPanos SoultanasDe LiesPantelis TopalisDe WilfredoPaola BarbaDeanna ArsalaPaola BellostaDebarati BasuPaola PiomboniDebasish DeyPaola PiscopoDeborah AgostiniPaola VittoriosoDeborah CharlesworthPaolina CroccoDeborah FinlaisonPaolo AbondioDeborah GoodPaolo Ajmone-MarsanDeepak SinghPaolo CinelliDeepanwita BanerjeePaolo FontanaDeepti NigamPaolo MariottiniDegeng WangPaolo PelliccioniDeirdre CampionPaolo RibecaDeisy GysiPaolo TarantinoDelphine GobertParaskevi KarousiDelphine LegrandParesh PrajapatiDemin CaiParis VeltsosDenis FirsanovParna SahaDenise HaroldParra Maria TeresaDenise MéthéParveen ShirazDennis McNevinPascal EscherDennis MillerPascale QuignonDerek MorrisPatricia KhashayarDerek WildmanPatricia Recordon-PinsonDerrick MathiasPatricia ToninDesalegn SerbaPatrick CarrollDevendra GuptaPatrick DiMarioDevin KirkPatrick EderyDezső MódosPatrick JayDhananjay PandeyPatrick M. WosterDhananjaya KulkarniPatrick MayDhruvajyoti RoyPatrick MerciéDi ZhangPatrick O’DonoghueDiaa DaghmaPatrick RichDianne NewburyPatrick Varga-WeiszDidier JollivetPatrick Vourc'hDiego BreviarioPatrik KällbackDiego CentonzePatrizia ProiaDiego CortezPatti MillerDiego HojsgaardPaul A. SievingDiego MastroeniPaul AirsDiego MoraPaul ArcieroDiego Peres-AlonsoPaul BatesDiego San MauroPaul CarrierDik Van GentPaul CouckeDimitra KaragkouniPaul GoldwaterDimitri PoddighePaul GrabowskiDimitrios AnyfantakisPaul HeathDimitrios ChatziplisPaul MischelDimitrios PapadimitriouPaul MorganDimitrios RoukosPaul R. CopelandDimitris LagosPaul ReynoldsDimitry SchepetovPaul SandstromDimosthenis KizisPaul SiegelDina ProestouPaul ValdmanisDino SamartzisPaula SoaresDiogo Cabral-de-MelloPaula WatersDipankar RoyPaulina BolivarDiptee ChaulagainPaulina Carmona-MoraDirk HecklPaulina Niedźwiedzka-RystwejDirk SchenkePaulina ŻarnowiecDirk SmitPavel KostylevDisha SharmaPavel MadonovDivya KalraPavel OstasovDivya RamchandaniPavla BrachovaDivya SahuPavle RandjelovicDivya SharmaPaweł AntosikDixie GossPaweł CięszczykDmitrii BugPaweł KordowitzkiDmitry DedukhPaweł LipińskiDmitry MukhaPawel SobczukDomenico AielloPedro Gonzalez-MenendezDomenico CovielloPedro LoriteDomenico LioPedro SeoaneDomenico PalumboPeer BergDominga SogliaPei-Fen YenDomingo González-LamuñoPeixin DongDominik DłuskiPeixin WuDominik KopczynskiPeng WeiDominik SaulPeng-Hui WangDominik SeelowPengxiang ChangDominik TomaszewskiPenny K. RiggsDominik WestphalPere PuigdomènechDominika SalamonPeter AndersenDominique De ViennePeter BoagDominique WagnerPeter De KnijffDonald ColganPeter DevileeDonat AlparPeter EllisDonata WawrzyckaPeter FalkaiDonatella MilaniPeter GillDonato GemmatiPeter KangDong LeePeter PikoDong ZhangPeter ScacheriDongchuan GuoPeter SeeberDoris Rosero-GarciaPeter Van HasseltDorota Fopp-BayatPeter WehlingDorota Iwaszkiewicz-GrzesPetr BuresDorota RowczenioPetr KotlíkDorothea ThompsonPetr SedlakDouglas CookPetr SmardaDouglas RootPetra JiroutovaDouglas WeiserPetros KolovosDov HershkovitzPetrus TangDragan MilenkovićPhilipp BrandDragan PrimoracPhilippe DeschampsDror SharonPhilippe GallusciDuangjai TungmunnithumPhilippe LabruneDubravka ŠtracPhilippe MongetDubravka Švob ŠtracPhilippe TanguayDukka K. C.Philippe VandenkoornhuyseDusana MajeraPhillip GroteDuy DoPhillip MccownDylan SosaPhillip SponenbergEckhardt ErikPhillip YatesEd BoltPhoebe ChenEd SmithPiergiorgio La RosaÉder PetryPiergiorgio ModenaEdinson Meregildo RodriguezPiero FariselliEdith HoferPierre-François CartronEdoardo BertoliniPierrick BourratEdoardo MalfattiPietro CacialliEduardo Ruiz-PesiniPietro GramazioEdward HigsonPilar SoengasEfstratios-Stylianos PyrgelisPinar TulayEishi NoguchiPinelopi ArtemakiEkaterina ChesnokovaPing YeEkaterina ZubarevaPing-Chieh PaoElad LaxPiotr BednarekElad OrenPiotr BialasiewiczElaine OstranderPlacido IllianoElaine YeungPo-Jen HsiaoElena AndreevaPolona Le Quesne StabejElena ApostolovaPontus ErikssonElena BelykhPrabhakar BastolaElena BitocchiPrabhakaran SoundararajanElena BonoraPrabhu MathiyalaganElena BoschPradip DeElena CampionePrafull SalviElena CianiPrakash VenglatElena De FelicePranshu SahgalElena Domínguez-GarridoPrapaporn Techa-AngkoonElena DubinaPrashant KaushikElena IliePrashi JainElena ObradorPrathap MahalingaiahElena OniciucPratyush RoutrayElena Ramos-TrujilloPraveen Raj KumarElena RampanelliPrimrose FreestoneElena SalviPriyanka KhareElena SarropoulouPriyanka KulkarniElena SeminaPriyanka MishraElena ShekhovaPrzemko TylzanowskiElena TenediniPrzemysław KołodziejElena ValeevaPrzemyslaw KosinskiElena ZaklyazminskayaPrzemyslaw SzafranskiEleni DouniPurva AbhyankarEleni LikotrafitiQi YanElenjikkal SirilQien YangElfride De BaereQihua TanElham RezaeiQing LiuElham SajjadiQinghang MengElia Benito-GutiérrezQinghua CuiEliana NutricatiQingyi YuElias AlisaacQingyu ZhouEliecer CotoQionghou LiEliezer GoldschmidtQiuwang ZhangElisa BindaQuan ZouElisa PorcelliniQuentin GouilElisabeth JonesQuentin SzaboElisabetta AntonelliQun ChenElisabetta BenedettiQunkang ChengElisabetta CilliQuynh NguyenElisabetta Di FedeR. ShanelyElisabetta TabolacciRaafat El-NamakyElise AlbertRachel GreenElissavet NtemouRachel Roberts-GalbraithElizabeth KramerRadhey GuptaElizabeth McMillanRadhey S. GuptaElizabeth PriceRadhia M’KacherEllen DecaesteckerRadka PourováEllen KnuepferRadka SymonovaElliot FriedmanRadomir SavicEllis O’NeillRadosław ChaberElmira MohandesanRadosław KempińskiElőd NagyRadovan KasardaElvira HörandlRadu-Stefan RomosanElvira ParrottaRafael JiménezElvira SpagnoloRafael KretschmerEmanuela SalzanoRafael Montalvo-RodríguezEmanuele D’AmicoRafael Salto-GonzalezEmanuele MicaglioRafaela VasiliadouEmanuele MondaRafail TasakisEmanuele RaineriRafe LyallEmil GustavssonRaffaele PezzilliEmil KrupaRaffaella CascellaEmilia BagnickaRaghavendra YadavalliEmília HanusováRagupathi NagarajanEmiliano GiardinaRahul SanawarEmiliano LasagnaRainer SpanagelEmiliano TesoroRajan AdhikariEmilija CvetkovskaRajendran SathishrajEmily De Los ReyesRajesh MisraEmma BerdanRajesh ParsanathanEmmanouil MalandrakisRajiv KumarEmmanuel ElangaRajiv MachadoEmmanuel TetaudRakmetkazhy BersimbaevEmmanuelle GéninRaktim RoyEmmanuelle NicolasRalf BraunEmre ErdemRalf BundschuhEnrico DoriaRalf Erik WellingerEnrique Medina-AcostaRalf-Erik WellingerEnrique MorettRalph SchlapbachEnriqueta GutierrezRam SagarEnriqueta Moyano CañeteRamanagouda BhojappaEran ElhaikRamcharan AngomEric B. KmiecRamesh PeriasamyEric BarreyRamesh RijalEric BeyerRamon AlmelaEric GalianaRamon AyonEric HendricksonRamon PerezEric HervouetRamyaa RamyaaEric JeandidierRamzi MohammadEric LintonRandall RoperEric LiuRandy DinkinsEric PageRanjith PapareddyEric PasmantRaphael PetegrossoEric ScholljegerdesRaquel AssisEric TothRaquel BernardinoEric VallenderRaquel DuranErica MackeRaquel VasconcelosErich Bornberg-BauerRaquel YahyaouiErich SchwarzRasa SabaliauskaitėErik BjörckRaúl Estévez PovedanoErik C. AndersenRaul J. CanoErik ErsmarkRaúl PérezErika CecchinRaúl Teruel-MontoyaErin AhnRavi MisraErin (Eun-Young) AhnRavindra KumarErnesto GomezRay ToblerErnesto PicardiRazmik MirzayansErwin BrosensRebecca KimballErwin GoldbergRebecca L. MickolEstefania Lozano-VelascoRebecca RancourtEstefania NuñezReema SinghEstelle Jumas-BilakRegie Santos-CortezEsther KadieneRegina Golan-GerstlEsther PomaresReidunn AalenEszter KapusiReilly KayserEto Silas FernandesReinhard KoppEtsuro OhtaReinhard UllmannEttore OlmoRéka MizseiEuan JamesRemo SangesEuan K. JamesRenake TeixeiraEugene BerezikovRenata FreitasEugenia DavidescuRendong YangEugenia MontielRené MarsanoEugenia PoliakovRenee ReamsEugenia SanchezReny MathewEugenio López-CorteganoRenzo MignaniEulalie LasseauxRex HessEustathios KenanidisRhiannon LloydEva BagyinszkyRhoderick ElderEva Kurtz-NelsonRicard AlbalatEva ŠatovićRicardo Cruz-LópezEva TemschRicardo Dinis-OliveiraEva TrevissonRicardo DuarteEva VarallyayRicardo LebrónEvan DurlandRiccardo CastigliaEvangelista LauraRiccardo IentileEvanthia BletsaRiccardo MorettiEveline Ibeagha-AwemuRicha BatraEveline PeetersRichard BinghamEvelise BrizolaRichard BolesEvelyn JensenRichard CordauxEvgenii SkurikhinRichard EspleyEvgeny ErmakovRichard IvellEvgeny ZakharovRichard LinnérEvzen KorecRichard Osei-AmponsahEwa Boniewska-BernackaRichard PaiseyEwa KuźnickaRichard ThompsonEwa Sell-KubiakRicia HydeEwelina MaculewiczRima SlimEwelina PerdasRita BaroneEwelina Semik-GurgulRita BonfiglioEwelina SzacawaRita ČepulienėEyal FridmanRita CowellEyal SeroussiRita FiorFabiana EspositoRita NunesFabien DelerueRob IllingworthFabien SauvetRob Van HoudtFabien SénéchalRobbee WedowFabienne RajasRobert BestFabio CoppedèRobert BroshFabio StossiRobert Bryson-RichardsonFabrice FleuryRobert ChristensenFabrice LejeuneRobert FuentesFabrizio DamianoRobert GrahnFabrizio LombardoRobert Hänsel-HertschFabrizio MafessoniRobert HarrisonFang ZhengRobert HertelFangqing ZhaoRobert JansensFanny Morice-PicardRobert JarretFarah AmmousRobert JerniganFarida TripodiRobert KelshFarnoosh Abbas-AghababazadehRobert KleszczFatih DemirRobert KoflerFatima CvrčkováRobert KortumFatima De PalmaRobert McFarlandFatima El KhalloufiRobert MillerFaustino MollinedoRobert MukiibiFavetta LauraRobert OnzimaFederica BrunoRobert PhilibertFederica De LiseRobert PyleFederica Di PalmaRobert ReddenFederica MontagneseRobert UncklessFederica SangiuoloRobert WaterhouseFederico G. HoffmannRobert WinqvistFederico HoffmannRobert ZinnaFelicitas PfeiferRoberta BergeroFelipe Gómez GómezRoberta De Souza SantosFelix BeaudryRoberta EspositoFelix ClanchyRoberta FraschiniFelix Javier Jiménez-JiménezRoberta La StarzaFelix LockerRoberta NapolitanoFelix LoosliRoberta ParisFélix MachínRoberta VenturellaFeng ChengRoberto CanitanoFeng LiuRoberto CastiglioneFeng XueRoberto De La FuenteFerdinando GulinoRoberto MariottiFerdinando SquitieriRoberto Martín HernándezFereydoun HormozdiariRoberto OleariFernando LealRoberto PiergentiliFernando RohanRoberto TuberosaFilipa ValeRoberto VitaFilipe PintoRobyn KosinskyFilippo CiabrelliRocío BautistaFilomena NapolitanoRodney A. LeaFilomena NigrisRodney GilbertFin MacraeRodney N. NagoshiFina Martínez-SolerRodney ScottFiona McCarthyRodolfo IulianoFleming MargaretRodolfo ZentellaFlorian FriedmacherRodriguez JoseFlorian FrugierRoel OphoffFolker MeyerRogelio Palomino-MoralesFortunato LonardoRoger MoragaFotis TsetsosRoger SawyerFranca FagioliRoger WilliamsFranca GueriniRohit GulatiFrancesc AvilesRohit KoloraFrancesca AntonacciRohit MagoFrancesca ClementinaRoland AkakpoFrancesca CucinottaRoland P. KuiperFrancesca De SantisRoland SchafleitnerFrancesca DuraturoRolf W. StottmannFrancesca GranataRoman WenneFrancesca JeanRomana VulturarFrancesca MariRomi Pena SubiràFrancesca PaganoRomina RomanielloFrancesca TuortoRómulo SobralFrancesco Di CarloRona StrawbridgeFrancesco MassariRonald MyersFrancesco NardiRory JohnsonFrancesco PeriniRosa FregelFrancesco RavaioliRosalind JohnFrancesco SartiniRosanna InturriFrancesco SorrentinoRosanna MarinoFrancesco SunseriRosario PeronaFrancieli ChassotRoser Gonzàlez-DuarteFrancine Perrine-WalkerRoser UrreiztiFrancis AlexanderRoss LillFrancisco Barrionuevo JimenezRossella TricaricoFrancisco CastilloRossella TuplerFrancisco CobosRossiana BojinovaFrancisco DiazRoy CostillaFrancisco García-CózarRoy MoncayoFrancisco Garcia-GonzalezRuben PetreacaFrancisco Javier Barrionuevo JimenezRubina TabassumFrancisco Navas GonzálezRui ChenFrancisco PintoRui FuFranco BassettoRuihong WangFranco TaroniRuitu LyuFrancois BonhommeRupert EckerFrançois ChevalierRupesh TayadeFrancois GirodonRussell HamiltonFrançois RousseauRute PereiraFrancois SpitzRyan SchmidtFrane PaicRyan YuenFrank BuchholzRyoko Machida-HiranoFrank ChervenakRyoma MorigakiFrank KoRyuji MorizaneFrank KramerSaba BattelinoFrank TakkenSabine BrandtFrank Van SteenbeekSabine KleinsteuberFrans CremersSabine StrehlFrans KooymanSabrina CarrellaFrantišek MarecSabrina StranoFrauke CoppietersSadegheh HaghshenasFrauke StankeSadjia BekalFred ChenSahra UygunFred GageSai ChittiFred WinstonSaid AmiriFrederic ChevalierSaida OrtolanoFrederick StoddardSaifudeen IsmaelFrederique PeronnetSairam RudrabhatlaFriedhelm PfeifferSajeesh KappacheryFuhua HaoSally LauFulvio CrucianiSalvador AliñoFulvio Della RagioneSalvador F. AliñoFulya TaylanSalvador MireteGabe HallerSalvador PérezGabina Calderón-RoseteSalvatore D’AnielloGábor NagySalvatore De BonisGábor RigóSalvatore ScaccoGabriel Anaya-CalvoSam AlsfordGabriela BodeaSaman ZeeshanGabriela LinSamantha A. BrooksGabriele RondoniSamantha DonsanteGabriele ToiettaSameer ChavanGabriella De LorenzisSameer KhanalGabriella HorvathSamir AlahmadGabriella LindgrenSamir BarghoutGael RoueSamuel HazenGaëlle PennarunSamuel MartinGaetana PaolellaSamuel Robert JeyasinghGaetano OdiernaSamuele BovoGaia AnelliSandip GhugeGaia ManninoSandosh PadmanabhanGajender AletiSandra CorreiaGali MaorSandrine ChamayouGalina RalduginaSandrine LauranceGanesan GovindanSandro BanfiGanesh Malli MohanSaneyuki KawabataGanesh SwamySang-We KimGang PengSanjiv NeupaneGarth EhrlichSantiago CepedaGary HardimanSantiago García-MartínezGary JohnsonSantiago RodriguezGary PehSantos AlonsoGáspárdy AndrásSantosh GhoshGaurav MehtaSara Rodríguez-AcebesGavin ChapmanSarah Adjei-FremahGayle FergusonSarah BarclayGeert PoelmansSarah FerriGelsomina MansuetoSarah GinoGemma MaSarah H. ElseaGenyi LiSarah McCoskiGeoffrey MeruSarah MortonGeorg DewaldSarah NewburyGeorg HildenbrandSarah SawyerGeorge C. TsengSarah WongGeorge DiCenzoSaranya VeluswamyGeorge DuncanSaravanan KolandaiveluGeorge FthenakisSareena SahabGeorge GarinisSargis KarapetyanGeorge KomisSarit AvraniGeorge MarkozannesSascha KupkeGeorge TavartkiladzeSascha SchäubleGeorge WeiblenSatheesh SengodanGeorgeta TruscaSatoshi FukuchiGeorgia KaidonisSatoshi IwakamiGeorgia RagiaSau CheungGeorgios KarrasSaul Herranz-MartinGeorgios V. GkoutosSawan JhaGerald SpäthSayaka MiuraGerald ThomsenScott BlankenshipGéraldine LootScott PrattGerardo CazzatoScott SchuylerGermain GilletSean P. PlaceGerson OliveiraSebastian DemydaGertrud Grilz-SegerSebastián Demyda-PeyrásGiada AmodeoSebastián E. Ramos-OnsinsGiada ZaniniSebastian GlattGiampietro SgaragliSebastian GranitzerGianluca EspositoSebastian KnagaGianluca OcchiSebastian KummerGianpiero ZamperinSebastian MedinaGihyun LeeSebastian RoeschGil AtzmonSebastiano LuridianaGilad GabaySebastien Ferreira CercaGino FornaciariSedigheh DelmaghaniGiordano LiberiSeiji MasudaGiorgia BatelliSeinen ChowGiorgio DieciSelvarangan PonnazhaganGiorgio MalpeliSenthil ThamilarasanGiorgio PerrellaSeon-Yong JeongGiorgio SandriniSepalika Baththana MudiyanselageGiorgio StantaSepand RastegarGiorgio VallortigaraSeppo Ylä-HerttualaGiorgio VingianiSerena AcetoGiovanni AmoriSerena CavalleroGiovanni AnnonaSerena DatoGiovanni ChillemiSerena LattanteGiovanni CorsoSergey FedotovGiovanni LaviolaSergey KolomeǐchukGiovanni LeviSergey KozyrevGiovanni MessinaSergey NikolaevGiovanni NeriSergey RazinGiovanni NigitaSergi PuigGiovanni ScambiaSergio BernardiniGirmantė JurkšienėSeth FrietzeGironi CristinaSeung AnGissella VasquezSeung LeeGiulia CuriaSéverine BärGiulia Di DalmaziShahrokh GhobadlooGiulia FrissoShailesh KumarGiulia RioloShane HegartyGiuseppe AgapitoSharda SinghGiuseppe Alberto PalumboSharmila GhoshGiuseppe AngelicoSharon SinghGiuseppe CampoSharon TamirGiuseppe CasconeShashi Shekhar SinghGiuseppe De PalmaShawn BurgessGiuseppe GiuseppeShay CovoGiuseppe GrazianoSheikh RiazuddinGiuseppe IacominoShekhar SahaGiuseppe LimongelliShekinah ElmoreGiuseppe LucarelliShelley AdamoGiuseppe MarangiSheng JinGiuseppe PalmisanoSheng WangGiuseppe PassarinoSheng YingGiuseppe SacconeSheng ZhuGiuseppe SiragoSheng-Fan WangGiuseppe Visani‪Shengqian XiaGiuseppina RoseShi Song RongGiusy TornilloShibiao WanGizella JahnkeShigekatsu SuzukiGleb ArtemovShigeki IwaseGloria BertoliShigeki KuriyamaGloria Del Solar DongilShihua ShenGloria Soberón-ChávezShikun HeGoel ManviShilpa ThakurGokhan CildirShin ShuGonzález-de SandraShinichi HochiGonzalo Solís SanchezShin-ichi HorikeGoran CuricShinichi TakaichiGordana ErjavecShinji AsanoGoutam SahanaShinobu MatsuuraGozde DemirerShinpei YamaguchiGracia Merino PeláezShinsuke FujiokaGraham WilliamsShipra GroverGraziano PesoleShirong LiuGraziella CappellettiShishir GuptaGrażyna PolakShisong RongGregg ThomasShizuka UchidaGregório De CamargoSho TsukamotoGregorio Moreno-RuedaShoichiro KokabuGregory BartonShrikant PawarGregory FindlayShuangping LiuGregory FraleyShu-Bing QianGregory HawkinsShuchi WangGregory JohnsonShu-Chun LinGregory PastoresShuhei SegamiGrigoris AmoutziasShuji MizumotoGrigory DianovShujin LuoGrzegorz CiesielskiShun MaekawaGrzegorz JuszczakSian Durward-AkhurstGrzegorz SmołuchaSibte HadiGuangchun HanSiddharth JaggavarapuGuanghua XiaoSiddhartha DasGudrun RappoldSiddhartha DebGuenther BuchholzSiew-Kee LowGuglielmina PepeSi-keun LimGuido GembilloSilvana BriugliaGuido J. FalconeSilvia AkutsuGuido VeitSilvia BistiGuillaume CourtoySilvia Del VecchioGuillaume PavlovicSilvia FasanoGuillermo GervasiniSilvia Martin-AlmedinaGuillermo GiovambattistaSilvia PampanaGuiomar Perez De NanclaresSilvia Stefania LongoniGung LeeSilvia TurroniGunn‐Guang LiouSilvia Vega-Rubín-De-CelisGuohao WangSilvio SalviGuojun DaiSimm StefanGuoliang LiSimon BlanchetGuozhi XiaoSimon JonesGurnett ChristinaSimon PfanzeltGustavo ZuñigaSimona Jurkovic MlakarH. Diego FolcoSimone PaliniH. Ulrich GöringerSimone PompeiHaifei HuSimone Riis PorsborgHaihui YeSimonetta FrisoHailan FengSimran MaggoHaiping HongSini SkarpHaizi ChengSmiljana GoretaHalie RandoSnæbjörn PálssonHamed KavehSneha GuptaHamid Alinejad RoknySo NakagawaHamid BeikiSofia BhatiaHamza MbarecheSofia CostaHana VydrovaSofia GabrielHanan SelaSohier YahiaHanhan LiuSolomon ChakHanna SigemanSom DevHanno SchmidtSonali NashineHans A. HofmannSonam KumariHans HasselbalchSonia GarciaHans LehrachSoong LeeHans-Jörg JacobsenSophie BerlandHanying ChenSophie CaldérariHao YuanSophie NicoleHao-Xun ChangSophie RasmussenHarald LahmSophie SunHardik KundariyaSoumyadev SarkarHardy RideoutSreepriya PramodHarold BosseSrinivasan SrilakshmiHarrys JacobSruthy AugustineHarshraj ShindeSsu-Wei HsuHartmut EngelsStavros NikolaouHaruhiko SugimuraStefan AstromHasan MehrajStefan BekiranovHassan DastsoozStefan EngelhardtHassen GherbiStefan HopplerHatem El-ShantiStefan LangHea-Young LeeStefan PetersHeba EbeedStefan ZielenHector EscrivaStefania BortoluzziHeebal KimStefania SavoiHegyi PéterStefania ZampattiHeidi A. TissenbaumStefanie LehnerHeidi HauffeStefano CastellanaHeidi StöhrStefano Giuseppe CaraffiHelder NunesStefano LonardiHelen J. WingStefano MarelliHelena ŠtorchováStefano MartellucciHelene FachimStefano MonaHelga MiguelStefano MonacoHelga TorielloStefano PascarellaHengyao NiuStefano PavanHengyu LuStefano StagiHenner BrinkmannStefano VoliniaHenning WackerhageStella GagliardiHenrike ZschachSten AnslanHenry HengStephan ChristelHenry WirthStephan ReinertHerbert MeltzerStephan RipkeHerman MaysStéphane CoupéHermann GrafensteinStephanie CemanHernan FolcoStephanie DuguezHerwig BachmannStéphanie Leclerc-MercierHidehisa TakahashiStephanie McKayHideki SugiiStephanie TobinHidemasa BonoStephanie WongHidenori SuganoStephen BeverleyHideo NishitaniStephen BushHideyuki TanabeStephen FicklinHimashu SinghStephen HartHirohide UenishiStephen PakHirokazu TakahashiStephen TsoiHiroki YokotaStephen WalshHiromi Kajiya-KanegaeSteven LarsonHiromi MatsumaeSteven PopoffHironaga KakoiSteven PullanHiroshi KannoSteven SmithHiroshi MiyanishiStuart KimHiroyoshi TanakaStyliani GeronikolouHiroyuki HoriSubbaya SubramanianHiroyuki KurataSudheer GaraHiroyuki MishimaSudhir PandeyHiroyuki MochizukiSugandha SaxenaHisahide NishioSuhas KharatHisakazu KatoSujoy GhoshHisashi TanakaSuk MaHisato YagiSukhvinder SinghHitoshi ArakiSuman ChowdhuryHo KimSuman DuttaHolger BudahnSumana ChintalapudiHolger WilleSumin LeeHolly LaVoieSumit ParikhHoma YazdiSumito DatekiHong ZanSung HongHong ZhengSung LeeHongchang CuiSung Sam GongHongjun WangSunghan KimHongliang HeSung-Min AhnHongmei JiangSungwon KimHongxu DongSunil SukumaranHongyan ChaiSuping ZhouHongyan XuSuresh KalathilHorst ZitzelsbergerSuresh PantheeHossein TaghizadehSusan CoortHotcherl JeongSusan LamontHouria DaimiSusan M. DownesHoward FescemyerSusana BalcellsHsien-Yuan LaneSusana Gómez-BarreroHsin-I. ChangSusana PereiraHsueh-Ping ChuSusana VingaHua LuSusanna CogoHuaijun ZhouSusanne KohlHubert SytykiewiczSusheel GunasekarHugo MartinianoSushil DubeyHugo Reyes-CentenoSusumu MitsuyamaHugo SoudeynsSuzanne McDermottHugues ChapSven Van IjzendoornHui ChenSvenja AlterHui JiangSvenja PossedaHui LiSvetlana GalkinaHui YeSvetlana UzbekovaHuichun XuSwapan ChakrabartyHuitong ZhouSwati JainHumaira GowherSwayam SrivastavaHuyen PhanSyed IslamHwal JeongSylvia Le DévédecHwan YoonSylviane MullerHyejung WonSylvie Tuffery-GiraudHyojoong KimSylwia CzarnomskaHyoun-Ah KimSylwia RzońcaHyuk LeeSzilvia KuszaHyun KimSzilvia VeresHyun KwunSzymon JanczarHyung KimSzymon KubalaHyun-Woo KimTaeok BaeIan CarrTaichiro GotoIan MacraeTakaaki HayashiIan O’NeillTakaaki ShimohataIan PaulsenTakafumi KatoIan PriorTakafumi YokotaIan WallaceTakashi KokudoIbrahim SayedTakashi MoriguchiIdan ShalevTakashi NomizoIdit MayaTakato KobaIgnacio Del CastilloTakayo OtaIgnacio FernandezTakayuki TsukubaIgnacio SchorTakeshi OnumaIgnazio La MantiaTakeshi ShimogiriIgor AurerTakiy BerrandouIgor DeynekoTakuma IshizakiIgor JakovcevskiTakuro NakagawaIgor LebedevTamar Ben-YosefIguchi TaisenTamás OrbánIhab YounisTanaka TeruyukiIkuo SuzukiTania Martins-MarquesIlaria GuerrieroTanima BoseIlda D’AnnessaTanja GruberIldiko RaczTanja Grubić KezeleIldus AhmetovTanveer AhmadIlias EleftherohorinosTanya PaullIlka HeinemannTao PanIlka Maria AxmannTao YangIlya AkberdinTao YaoIlya Gavrilov-ZiminTara CarthyImeke GoldschmidtTarek ElghetanyImtiaz RandhawaTaslima HaqueIndrayani WaghmareTasneem SharmaIneke Van Der BurgtTássio De OliveiraInes HeilandTatiana DeniskovaInga ProkopenkoTatiana KulakovskayaIngo EbersbergerTatiana ShelengaIngo SchubertTatsuhiko GotoIngrid HedenfalkTea TomljanovićIngrid HolmTécher HervéIngrid OlesenTed BrownIngrid SpiesTe-Hua HsuIngrid TonhajzerovaTejas BouklasInken WohlersTeka KhanInmaculada Barranco-ChamorroTeng LiInmaculada CuchilloTerenzio CosioInmaculada Garcia-RoblesTeresa ValencakInmaculada Martín-BurrielTerje RaudseppIno CurikTerrence FureyIoannis EleftherianosTerry HaganIoannis KyriakidisTerry-Elinor ReidIoannis TsakmakidisTerry-Lynn YoungIoannis VamvakarisTetsufumi TakahashiIoannis ZaganasThane PapkeIoannis-Marios DiamantopoulosThangavel SamikkannuIon MandoiuThangiah GeethaIrena MladenovaThemis GiannoulisIrena SmagaTheo DreherIrena WaleckaTheocharis KonstantinidisIrena Wojsyk-BanaszakThierry MagnaldoIrene BottilloThierry WirthIrene MadrigalThinh ChuIrene RicciThomas A. CiullaIrene StefaniniThomas BartnikasIrene Vázquez-DomínguezThomas BeckingIrina BakloushinskayaThomas BlankersIrina BogolyubovaThomas BottonIrina GolovlevaThomas CaspariIrina MateiThomas EggermannIrina VelskoThomas FalguièresIrina YushenovaThomas FleischerIris BiebachThomas FriedmanIrmgard Irminger-FingerThomas GreitherIsabel FilgesThomas KlopstockIsabel HenriquesThomas KuhlmanIsabel MartinsThomas LiehrIsabel SierraThomas LopdellIsabella CeccheriniThomas MüllerIsabella ParoliniThomas MürdterIsabelle HenryThomas NiehausIsabelle QuéréThomas NussbaumerIsabelle RouxThomas OlsonIsabelle ThiffaultThomas OrtonIsabelle VachierThomas SmolIsidora RadulovThomas StoegerIslam RafiadThomas Van Overeem HansenIstván AndóThorarinn GudjonssonIstvan NagyThorsten AllersIstván PappTibor KristianIsura NagahageTibor TörökItalia Di LiegroTika AdhikariItziar EstensoroTill AdhikaryIulia ChiciudeanTilman Sanchez-ElsnerIván CsanakyTim Van DammeIvan FilhoTimothy CoxIvan Gomez-MestreTimothy HearnIvan LopezTimothy Sterne-WeilerIvan PejićTina BeyerIvan RadosavljevicTina ParadzikIvana BilicTing LiIvana JaricTing ZhangIvana NovakovicTingyu ChangIvana PeranTino PolenIvana ViolaTiphaine MartinIvonne LoefflerTitia SijenIwona Ben-SkowronekTiziana PandolfiniIwona GolonkaTobias GorisIwona RzeszutekTobias SkillbäckIwona Ziółkowska-SuchanekTod FullstonIzawa-Ishizawa YukiTodd ScheetzJ. Antonio BaezaTohru IkedaJ. Joe HullTom FlemingJ. Peter GogartenTom GoldammerJ. Peter W. YoungTomaiuolo RossellaJaakko SarparantaTomás Ripoll-VeraJackson ChamperTomasz KluzJacky YeungTomasz LitwinJacob DayTomasz StrzalaJacob JosephTomasz SzwaczkowskiJacqueline BatleyTomasz Za̧bekJacqueline DoyleTomasz ZokJacqueline SmithTomer Avidor-ReissJacquelyn EvansTomislav MeštrovićJacques ChomilierTomohiko KazamaJae BahnTomoki KoshoJae Yong HanTomoko UeharaJae-Heung KoTomonari KinoshitaJaeson CallaTomoyoshi NozakiJae-Won HuhTomoyoshi TeradaJae-Yeol JooTong LiuJaeyoung ChoiTong-Hong WangJagdeep WaliaTongwu ZhangJagoda PlaczkiewiczTony GambleJaime Bustos-MartínezTony HollandJair Tenorio-CastañoTorben RedmerJakub CieślakTorsten ThalheimJakub RokToshifumi TsukaharaJames AndersonToshifumi YokotaJames BrasicToshihiko EkiJames CampanellaToshiyuki SetoJames CoffmanTossaporn SeeherunvongJames CohenTosso LeebJames MalletToyoko AkiyamaJames MickelsonToyomasa KatagiriJames RiordanToyota KenjiJames SelfTraci MarinJames Van EttenTracy BrooksJan De VriesTrevor RomsdahlJan EngelhardtTrine BildeJan FílaTrine JorgensenJan HaasTripti SharmaJan KammengaTriston HooksJan Krzysztof NowakTsutomu ShimuraJan NovákTuire KuusiJan PadekenTyAnna LovatoJan PithaTychele TurnerJan ŠevčíkUddalak MajumdarJan TkadlecUgo AlaJana LunerovaUgo BorelloJanani ParameswaranUimook ChoiJane DaviesUlrich JuliusJane StewartUlrich KellnerJane WelshUmberto MalapelleJane WilliamsUmberto TarantinoJanna KoppeUnni GrimholtJannick NiortUri GophnaJanusz GodlewskiUrs HeilbronnerJaquelin DudleyUrska CvekJaqueline Carvalho De OliveiraUrszula CytlakJared TalbotUte RadespielJaroslav DolezelUtkarsh KohliJaroslav SterbaVadim LebedevJarosław BurczykVaibhav JainJasenka WagnerValentina G. KuznetsovaJasmin LalondeValentina GinevičienėJason BazilValentina MurdicaJason FlannickValentina RiggioJason HeValeria ContiJason MearsValeria D’ArgenioJason P. WeickValeria Di DatoJaspreet SharmaValeria VisconteJavier AvalosValérie De Crécy-LagardJavier CorralValérìe GeffroyJavier MartinValerie Odero-MarahJaya AseervathamValerie O’LearyJaya PadmanabhanValeriia HaberlandJayakumar NairVanessa LaaksonenJe LeeVânia CamiloJean MolinierVarunseelan MurugaiyanJean-Baptiste Le PichonVasia MavrogianniJean-Baptiste VannierVasile FlorianJeanette ErdmannVasileios StathiasJean-François PicimbonVasileios ToulisJean-Lou JustineVasiliki PanagiotakopoulouJean-Luc WautierVasiliki PappaJean-Michel RozetVasily AshapkinJeannette BensenVenera TyukmaevaJeehun LeeVenkat MalladiJeevisha BajajVenkatesh ThirumalaikumarJeff ColeVenkateswara GogulamudiJeffery DahlbergVerena PichlerJeffrey FawcettVeria VacchianoJeffrey FederVernazza StefaniaJeffrey KiddVeronica Risco-CastilloJeffrey P. MowerVeronika BensonJeffrey PaullVeronique GiudicelliJeffrey StuartVicki ThompsonJe-Ho MunVicky WhittemoreJe-Keun RheeVictor AsensiJelena Korac PrlicVíctor J. CidJennifer A. LeonardVictor JinJennifer BrunoVictor RomanovJennifer CihlarVictoria SleightJennifer JohnstonVidyalakshmi SethunathJennifer Taylor-CousarViera KaraffovaJennifer ThomsonViera SchwarzbacherováJens SchusterVijayaprakash SuppiahJeonghwan SeoViktor TsyganovJeppe AndersenVincent HascallJeremy CoateVincent PagèsJeremy EgbertVincent ReyesJeremy GuggenheimVincent Van RompaeyJeremy MurrayVincenzo L’ImperioJeremy SearleVincenzo MarottaJeroen HugenholtzVincenzo NigroJérôme RobinVincenzo ZappavignaJeronimo BravoVinesh VinayachandranJessica FortinVinit ShanbhagJessica HaywardVinita ChittoorJessica LubellVinod TiwariJessica MandrioliVirág ÁcsJessica RanieriVirag SharmaJesus BaroVirgilio SacchiniJesús FernándezVirginie VerhoevenJesús HuertasVishal GohilJesús PalomeroVishal KhivanasraJesús VielbaVishnu TripathiJi HuangVitaly KozinJia SongVito GuarnieriJiafu TanVito TerlizziJialei DuanVittoria DisciglioJian HuangVittorino NovelloJianbin SuVittorio De FranciscisJianbo YaoVivek BhardwajJianke LiViviana BarraJian-Min ChenViviana MoresiJianming WuViviana VallacchiJianping XuVlad PădureanuJianqiu XiaoVladimir BabenkoJianShe WangVladimir ChoobJianzhou CuiVladimir FilkovJia-Yu ChenVladimir GokhmanJie WangVladimir KorinekJie YangVladimir LazarevićJi-Hyeon JeonVladimir StrelnikovJikui SongVladimir VladimirovJin JeoungVladislav ČurnJin ParkVolkan ErginJindřich ChrtekVolker GurtlerJindrich CitekVolodymyr RadchukJing PengW. Allan KingJingjing YangW. WardJinhee LimWade BerritinniJinming HanWai ChuJinu HanWaleska DornasJin-Yong LeeWalter DoerflerJiqiang (Lanny) LingWalter KnightJiri KoreckyWalther ParsonJitendra KanaujiyaWalther TrautJitendra KumarWang-Tso LeeJiuzhou SongWankun XieJo GiaconiWarren SnellingJoachim AltschmiedWayne KnibbJoachim KrebsWei KongJoana CoutoWei LeiJoana DamásioWei WangJoana PintoWei ZhangJoana R LoureiroWeihua HuangJoann ConnerWeihua TianJoanna JaworskaWei-Jen ChenJoanna MelonekWeikun XiaoJoanna RogWeili DiJoanna WróblewskaWei-Li DiJo-Anne BrightWei-Ming LiJoão CardeiraWeirong XingJoao MeidanisWeizhen LiuJoao TeixeiraWellison Da Silva DinizJoaquin NavascuesWencheng LiJocelyne DiRuggieroWenhui SuJochen ImigWen-Juan MaJochen PrehnWen-Lii HuangJoel CorushWen-Ling LiaoJohan LedinWenndy HernandezJohann SchernthanerWenshih TsaiJohanna ElsensohnWijeratne AselaJohanna UngerstedtWilbert VermeijJohanne BrooksWilfredo De Jesús-RojasJohannes BirtelWillem StockJohannes HoferWilliam BrunoJohannes LeiererWilliam J. MurphyJohannes RudolphWilliam MarzluffJohannes StraussWilliam NewmanJohansson PatriciaWilliam SchierdingJohn BrigandeWilliam SchwanJohn ElefteriadesWilly BaarendsJohn GatesyWilnelly Hernandez-SanchezJohn GriggWioletta BielJohn H. PostlethwaitWioletta Sawicka-ZugajJohn LaiWipawee WinuthayanonJohn LindoWojciech BaranJohn NgunjiriWojciech KozdruńJohn NitissWolfgang ForstmeierJohn PappasWolfgang LinkJohn PostlethwaitWolfram S. KunzJohn ReaganWonchung LimJohn SoghigianWon-Sik HamJohn WelchWoo-keun KimJohn ZaiaXenia GondaJolanta Behnke-BorowczykXénia LatypovaJolanta ChmielowiecXi ChenJonas PaulsenXia YangJonas WixnerXiang ChenJonathan ColemanXiang YeJonathan KellerXiang ZhaoJonathan RiosXiang-Yang LouJonathan SeidmanXiao WangJonathon MohlXiaobo DongJong KimXiaodong ZhangJong LeeXiaohu HuangJongki ChoXiaolei LiuJoong-Ki ParkXiaoli PingJordi RipollXiaoli ZhangJordi SalmonaXiaoling ZhangJordi TamaritXiaona YouJörg FuchsXiaoqian ChangJörg StehleXin LiJorge CalvoXin XuJorge RochaXingguang LuoJorge VieiraXingming DengJoris BertrandXingxing WangJoris DeelenXinzhuan SuJoris VeltmanXiphias ZhuJoris VermeeschXu PengJosé A. MercadoXu WangJosé Antonio Garrido-CárdenasXuan LiJose FontenlaXuan ZhuangJose Lopez-EscamezXuan-Hung HoJosé M. LeitãoXuankun LiJosé María Martínez-De-La-CasaXuegang YuanJose María RequenaXueqin GaoJose Maria Vinardell GonzálezXylena ReedJosé Molina-MoraYaddanapudi RavindranathJose MontullYadong WangJose Moruno ManchonYakov DemurinJose PuzoYalda JamshidiJose QuilezYali SunJose RequenaYan GuoJosé RueffYang DaJose-Antonio Pedroza-GarciaYang ZhaoJosee Guirouilh-BarbatYanhao LaiJosef FinstererYanjiao ShaoJosef FulkaYann HéraultJoseph D. BuxbaumYannick D. BenoitJoseph KochmanskiYanrong QianJoseph McMillanYao YaoJoseph MianoYaroslav TeperJoshua EdwardsYassine SouilmiJosip TomićYasuaki KakinokiJovan PesovicYasukazu DaigakuJoy MitraYasuko IshidaJuan Alberto Marchal OrtegaYasushi ShiomiJuan C. OliverosYasuyuki FukuharaJuan CadiñanosYawei LiJuan Carlos Izpisua BelmonteYe GaoJuan Del CosoYei LaiJuan Francisco MartínYichuan LiuJuan ImperialYi-Fan ChenJuan José Pasantes LudeñaYijun PanJuan Mejía-AranguréYing ChanJuan Pérez-MorenoYing PangJuan PericasYingdong ZhaoJuan SantosYingJu LaiJuan Serrador PeiróYino WuJuan SubiranaYoav AravaJuan-Jose ArranzYoichi HondaJudit VárkonyiYoko MatsudaJudith ArmstrongYoko SattaJudith DavieYolanda Espinosa-ParrillaJudith FischerYong-Duo SunJudith HaendelerYongfeng ZhouJudith HallYong-jie XuJudith Klein-SeetharamanYongjin YooJules FreemanYongkang YangJulia HorsfieldYongqiang WangJulia MetzgerYongyong ShiJulia VainonenYongzhen HuangJulie DemarsYoon LeeJulie Guillermet GuibertYordan MuhovskiJulie HicksYork MarahrensJulie SardosYoshihiro FukumotoJulie SecombeYoshihiro KishidaJulie UrbanYoshihiro YamaguchiJulien DairouYoshihisa IkedaJulien RougeotYoshimitsu FujiiJuliette RoelsYoshiyuki KuroiwaJulija ErhardtYoshiyuki OgataJulio AguadoYoshizaki KaichiJulio EscribanoYosi MaruvkaJulio Sáez-VásquezYosra FalfoulJun AidaYoung Chang SohnJun CaiYoung KimJun GojoboriYoung ShinJun HosoeYoung SongJun SatoYoung YounJun SeoYoung-zoon KimJun TakeuchiYoun-sung KimJun UedaYousuke NakaiJun YuYousuke TakaokaJune CarrollYuan-Chuan ChenJung KimYuchin LienJung-Kyoon ChoiYue ShengJung-Wook KimYue WanJunil KimYuefei JinJunjie ZouYuhong TangJunming YueYu-Ichi GotoJunya TomidaYuichiro WatanabeJunzhong LiuYujiao LuJurg OttYukihiro NagashimaJürgen EngelYulema ValeroJürgen FrölichYuliang WuJustin CotneyYuliya VeryaskinaJustin GreerYun-Fai Chris LauJustin KumarYunfeng XuJustin MillerYung-Chih ChenJustin SzotYung-song WangJustyna PaprockaYun-Gui YangJyotsna BatraYunhao WangKacper ŻukowskiYuning ChenKai GuoYunting PuKai KisandYun-Zheng LeKai YuYuri A. MotorinKali MakedouYuri BattagliaKalliopi ZachouYuri LebedevKamalakar ChatlaYuri NesmelovKamalika MojumdarYuri PirolaKamel Abd-ElsalamYuri TrusovKamel LaghmaniYuriy OrlovKamil GillYury LoikaKamila Kusz-ZamelczykYutaka SuzukiKamila NowosadYuting GuanKamran ShazandYuu HiroseKanae NishiiYuuta MoriyamaKanai MasatakeYuval ItanKaren AvrahamYuyoung JooKaren CrawfordYvan DevauxKaren GronskovYves HenryKaren GrønskovZ. SoranKaren HeZacharias KontarakisKaren M. VasquezZachary BurtonKaren OverallZachary TaylorKaren SalazarZafar HandooKarin KirschnerZakariya AlgamalKarl DuderstadtZamora Ana MaríaKarl RavetZbigniew AdamiakKarl RobinsonZbigniew JablonowskiKarol Rawicz-PruszyńskiZdravko PetanjekKarolina Nowicka-BauerŽeljko AntićKarolina PierzynowskaZemfira KaramyshevaKároly MárialigetiZengdi ZhangKartik AnandZengjian Jeffrey ChenKasavajhala PrasadZenkong DaiKasturi PalZenon PidsudkoKatarina Trebušak PodkrajšekZeynep TümerKatarzyna KuterZhaohui S. QinKatarzyna MorkaZhaoning WangKatarzyna NucZhaoqi WangKatarzyna Pachulska-WieczorekZhenbin HuKatarzyna PiórkowskaZhenchang LiangKatarzyna RobaszkiewiczZhenghan WangKatarzyna Ropka-MolikZhenhui ZhongKatarzyna SikorskaZhenna XiaoKatarzyna WadowskaZhenqiu LiuKate TsaiZhenyu HuKaterina GrafanakiZhi ZhengKaterina KomrskovaZhipeng LiuKaterina PsarraZhiyong LiuKatharina HöferZhiyu YangKatherine FantauzzoZhongde WangKathleen PreißlerZhonglin TangKathryn WeaverZhousheng XiaoKathryna RodriguesZhu ZhuoKatia Del Rio-TsonisZihua HuKatiuska González-ArzolaZiliang LuoKatja EcklZippora BrownsteinKatja SchlattererZiru LiKatrina MorrisZissis MamurisKatrine JohannesenZofia MadejaKatsunori HatakeyamaZoltan HeroldKatsushi TokunagaZoltán HeroldKaushik PandaZoltan IvicsKaustubh AdhikariZoltán MagyarKaveh Samimi-NaminZsolt TothKavitha RaoZsuzsanna BereczkyKawaguchi NanakoZsuzsanna LichnerKay NieseltZsuzsanna SzalaiKazama YusukeZubeda SheikhKazimierz KuliczkowskiZuoxu Xie


